# The healthy eating index 2020 and colorectal cancer risk: a prospective study based on the PLCO cohort

**DOI:** 10.3389/fnut.2026.1793362

**Published:** 2026-06-11

**Authors:** Huang Li, Linguo Shen, Ziyao Zeng, Yi Xiao, Dazhan Feng, Linglong Peng, Ling Xiang, Yuxiang Luo, Yaxu Wang, Hongke Cai

**Affiliations:** 1 Department of Gastrointestinal Surgery, The Second Affiliated Hospital of Chongqing Medical University, Chongqing, China; 2 Center of Urology and Nephrology, The Second Affiliated Hospital of Chongqing Medical University, Chongqing, China; 3 Department of Clinical Nutrition, The Second Affiliated Hospital of Chongqing Medical University, Chongqing, China; 4 Thoraxcenter, Department of Cardiology, Cardiovascular Institute, Erasmus University Medical Center, Rotterdam, Netherlands

**Keywords:** cancer prevention, colorectal cancer, diet quality, epidemiology, healthy eating index

## Abstract

**Background:**

The Healthy Eating Index (HEI-2020), a metric that assesses overall diet quality based on compliance with the *2020–2025 Dietary Guidelines for Americans*, has been insufficiently investigated in relation to both colorectal cancer (CRC) incidence and mortality. To address this gap, we conducted a population-based prospective study of 101,709 U.S. adults from the Prostate, Lung, Colorectal, and Ovarian (PLCO) Cancer Screening Trial, examining the associations between HEI-2020 adherence and risks of CRC incidence and death.

**Methods:**

A total of 101,709 participants were included in this study. Dietary information was collected using the Diet History Questionnaire (DHQ), and adherence to the HEI-2020 was assessed. Higher HEI-2020 scores indicated better adherence to a healthy diet. Cox proportional hazards regression analyses were performed to examine the association between HEI-2020 scores and CRC risk. To further explore the dose–response relationship between HEI-2020 scores and CRC incidence, a restricted cubic spline (RCS) model was applied. Subgroup analyses were conducted to identify potential modifiers that might interact with HEI-2020 in relation to CRC incidence, and sensitivity analyses were performed to assess the robustness of the established association.

**Result:**

This study included 101,709 participants, with 1,100 incident CRC cases and 314 deaths. The fully adjusted model showed that participants in the highest HEI-2020 quartile had a lower point estimate for CRC incidence than those in the lowest quartile, although this comparison did not reach statistical significance (HR = 0.86; 95% CI: 0.73–1.02). A significant decreasing trend in CRC incidence risk was observed across HEI-2020 quartiles (P for trend = 0.021). Higher HEI-2020 scores were significantly associated with lower CRC-specific mortality (Q4 vs. Q1: HR = 0.61; 95% CI: 0.45–0.85; P for trend = 0.001), and this association remained consistent in competing-risk analyses. Site-specific analyses suggested lower risks for distal colon cancer and a borderline lower point estimate for rectal cancer, as well as a decreasing trend in proximal colon cancer mortality. Sensitivity and subgroup analyses confirmed the robustness of the findings.

**Conclusion:**

This study suggests that higher HEI-2020 scores, reflecting better alignment with the Dietary Guidelines for Americans, are associated with a significant decreasing trend in CRC incidence risk and a lower risk of CRC-specific mortality.

## Introduction

Colorectal cancer (CRC) is the third most commonly diagnosed cancer and the third leading cause of cancer-related death in both men and women in the United States (1). In 2025, it is projected that 154,270 new cases will be diagnosed in men and 107,240 in women, with an estimated 52,900 deaths from CRC. The development of CRC is a slow process, providing opportunities for early detection and prevention (2). Risk factors for CRC include both genetic and environmental factors, with diet being one of the most significant modifiable risk factors, particularly in Western countries (3, 4). With an increasing incidence, especially among younger adults, CRC is expected to continue rising globally, highlighting the importance of preventive strategies (1). Given its strong link to diet, lifestyle interventions that promote healthier eating habits could be key in reducing the burden of CRC (4).

Epidemiological studies have shown that specific dietary factors may influence the risk of CRC. For instance, higher consumption of red and processed meats has been associated with an increased risk of CRC, as demonstrated by a study from the UK Biobank (5). On the other hand, a meta-analysis by Schwingshackl et al. (6) found that a greater intake of vegetables, fruits, and whole grains was inversely correlated with CRC risk. Furthermore, evidence from a systematic review suggests a protective role of fish consumption in reducing CRC risk (7). Additionally, a study of two US cohorts emphasized that increased intake of sugar-sweetened beverages was linked to a higher risk of early-onset CRC in women (8). While these studies predominantly focus on individual foods or food groups, they may not provide a complete understanding of an ideal dietary pattern for overall health. A more comprehensive approach, such as dietary patterns that include a variety of foods and nutrients, could offer a better perspective on diet’s role in CRC prevention. This highlights the importance of the Healthy Eating Index (HEI-2020) as a more holistic tool for evaluating diet quality.

The Healthy Eating Index (HEI) is a standardized tool developed by the U.S. Department of Agriculture (USDA) to evaluate the overall quality of an individual’s diet based on adherence to the Dietary Guidelines for Americans (DGAs), independent of total caloric intake. The HEI-2020, consistent with the 2020–2025 DGAs, includes 13 components that capture key aspects of dietary intake, such as fruits, vegetables, whole grains, dairy, protein sources, fats, sodium, and added sugars. The index allows for comparison across individuals with different energy intakes by evaluating diet quality on an energy-density basis (9, 10).

Based on previous research, higher scores on the HEI, which assess adherence to the Dietary Guidelines for Americans, have been associated with a lower risk of non-communicable diseases. Meta-analytic evidence indicates that higher HEI scores are linked to reduced all-cause mortality, cardiovascular disease incidence and mortality, cancer incidence and mortality, and type 2 diabetes incidence (11). In prospective cohort analyses, greater adherence to HEI-2015, reflecting conformity with the 2015–2020 Dietary Guidelines, has been associated with lower risk of cardiovascular disease events, reduced cardiovascular mortality, and decreased all-cause mortality after adjustment for demographic and lifestyle covariates (12). Cross-sectional studies using data from the National Health and Nutrition Examination Survey (NHANES) have shown that higher HEI-2015 scores correlate with lower predicted 10-year cardiovascular risk and more favorable cardiometabolic profiles in adult populations (13). In addition, observational studies have reported inverse associations between HEI scores and the risk of certain cancers. A case–control study found that higher HEI-2015 scores were associated with reduced breast cancer risk (14), and another multicenter analysis reported that higher HEI scores were associated with lower risk of oral and pharyngeal cancer (15).

To date, research focusing on the relationship between HEI-2020 adherence and CRC risk remains limited. Therefore, we conducted this analysis to examine the association between HEI-2020 adherence and CRC risk in 101,709 participants aged 55 to 74 years from the Prostate, Lung, Colorectal, and Ovarian (PLCO) cohort.

## Methods

### Study design

**Figure 1:**
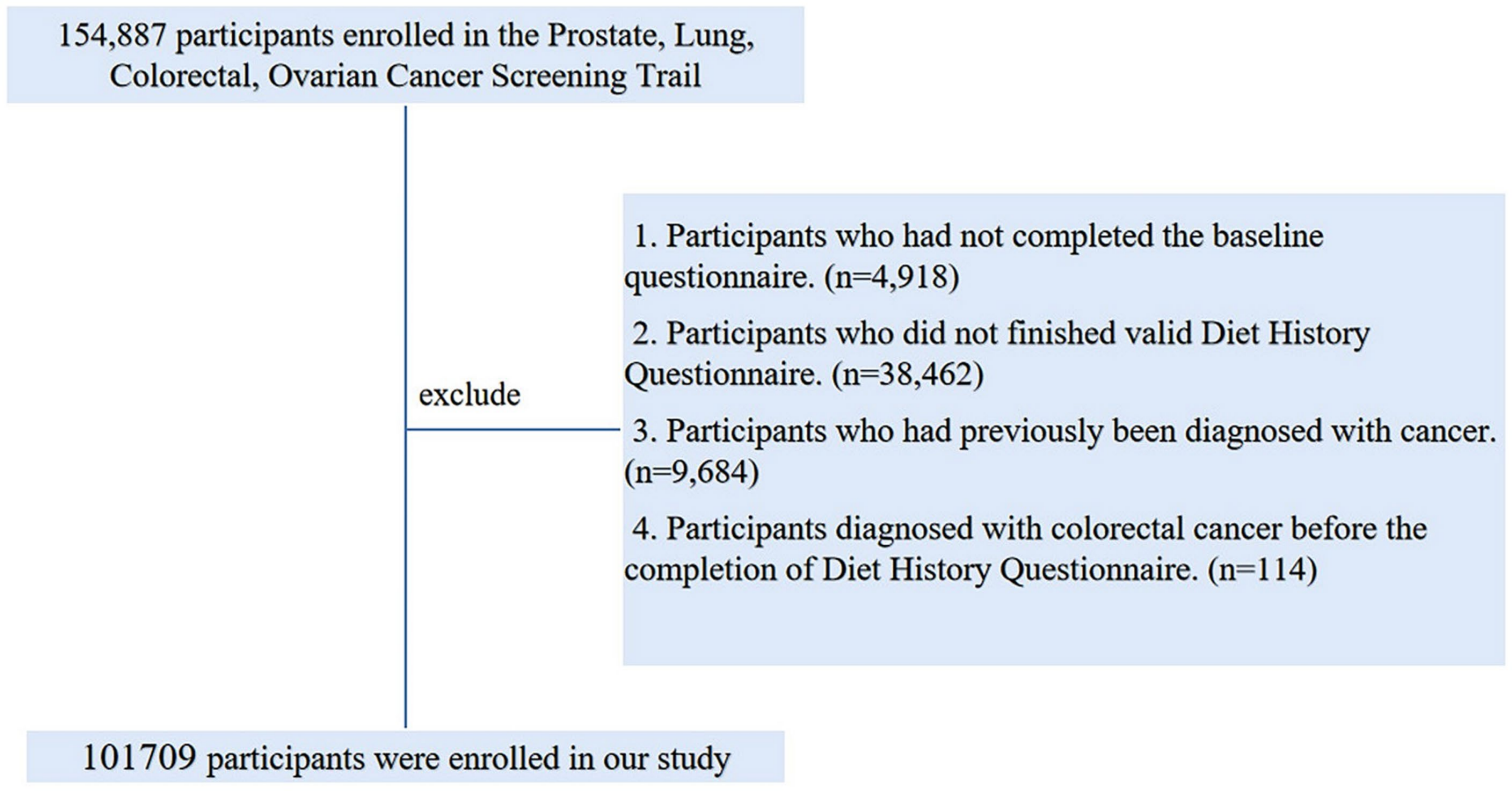
The flow chart of identifying eligible subjects. PLCO, Prostate, Lung, Colorectal, and Ovarian; BQ, Baseline questionnaire; DHQ, Diet history questionnaire.

The PLCO Cancer Screening Trial is a large, multicenter, randomized controlled study designed to assess the efficacy of screening methods for prostate, lung, colorectal, and ovarian cancers (16). Detailed descriptions of the trial have been provided elsewhere (16, 17). Between 1993 and 2001, 154,887 individuals aged 55 to 74 years were enrolled at 10 selected screening centers across the United States. Participants were randomly assigned in a 1:1 ratio to either the intervention arm or the control arm. Participants in the control arm received usual care, whereas those in the intervention arm were offered protocol-specified cancer screening examinations. For colorectal cancer screening, the intervention arm included flexible sigmoidoscopy at baseline and a repeat screening examination at either year 3 or year 5 according to the PLCO screening protocol. The control arm was not assigned PLCO protocol-based colorectal screening and continued to receive usual medical care (16, 17). At baseline, participants were administered some self-reported questionnaires, such as the Baseline Questionnaire (BQ), Supplementary Questionnaire (SQX), and Diet History Questionnaire (DHQ), to collect individual characteristics, including diet and other cancer risk factors. All screening procedures and individual medical record abstracting were performed by trained and certified specialists, and the cause of death was certified by the Death Review Committee (DRC). The PLCO Cancer Screening Trial was approved by the National Cancer Institute (NCI), one of the components of the National Institutes of Health (NIH), and each of the 10 screening centers involved in the study, all participants provided explicit, informed, and written consent (18). Our research was carried out with the approval of the NCI (project number: PLCO-1886). In consideration of the objective of our study, we further excluded subjects as follows: 1. Failure to complete the BQ (*n* = 4,918); 2. Invalid DHQ responses were defined as failure to return the DHQ, lack of a completion date, DHQ completion after the death date, a high frequency of missing responses (≥8), or extreme total energy intake according to the PLCO DHQ validity criteria (n = 38,462). Extreme total energy intake was identified using the PLCO DHQ extreme-energy flag, which marks the sex-specific lowest and highest 1% of total energy intake. In the source DHQ dataset, the corresponding thresholds were ≤552.62 or ≥5619.84 kcal/day for men and ≤466.11 or ≥3685.92 kcal/day for women. These cohort- and sex-specific thresholds were used because they were part of the PLCO DHQ validity criteria and reflected the dietary intake distribution within the study population; 3. Have history of cancer prior to DHQ administration (*n* = 9,684); 4. Participants who exited from the PLCO cancer screening trial between enrollment and DHQ completion (due to outcome events, death, or loss to follow-up) (*n* = 114). Finally, as illustrated in Figure 1, a total of 101,709 participants met the inclusion criteria, comprising 52,250 females and 49,459 males.

### Healthy eating index scores assessment

The HEI-2020 was used to assess overall diet quality according to the 2020–2025 Dietary Guidelines for Americans. Dietary information was obtained from the DHQ, which assessed participants’ usual food and nutrient intake over the preceding year. The HEI-2020 consists of 13 components, including nine adequacy components and four moderation components. The adequacy components include total fruits, whole fruits, total vegetables, greens and beans, whole grains, dairy, total protein foods, seafood and plant proteins, and fatty acids. The moderation components include refined grains, sodium, added sugars, and saturated fats (9, 10).

Component scores were calculated according to established HEI-2020 scoring standards. For adequacy components, higher intakes receive higher scores, whereas for moderation components, lower intakes receive higher scores. Most components are scored using an energy-density approach, such as amounts per 1,000 kcal or percentage of total energy intake, to reduce the influence of total energy intake. The total HEI-2020 score was obtained by summing all component scores and ranges from 0 to 100, with higher scores indicating better adherence to the 2020–2025 Dietary Guidelines for Americans. The scoring standards are shown in Table 1 (9).

**Table 1:** Healthy Eating Index (HEI)-2020 components and scoring standards ^a^.

Component	Maximum points	Standard for maximum score	Standard for minimum score of zero
HEI-2020 Applies to ages 2 and over			
Adequacy components			
Total fruits ^b^	5	≥ 0.8 cup equiv. per 1,000 kcal	No Fruit
Whole fruits ^c^	5	≥ 0.4 cup equiv. per 1,000 kcal	No Whole Fruit
Total vegetables ^d^	5	≥ 1.1 cup equiv. per 1,000 kcal	No Vegetables
Greens and beans	5	≥ 0.2 cup equiv. per 1,000 kcal	No Dark Green Vegetables or Legumes
Whole grains	10	≥ 1.5 oz. equiv. per 1,000 kcal	No Whole Grains
Dairy ^e^	10	≥ 1.3 cup equiv. per 1,000 kcal	No Dairy
Total protein foods ^d^	5	≥ 2.5 oz. equiv. per 1,000 kcal	No Protein Foods
Seafood and plant proteins ^f^	5	≥ 0.8 oz. equiv. per 1,000 kcal	No Seafood or Plant Proteins
Fatty acids ^g^	10	(PUFAs ^h^ + MUFAs^i^)/SFAs ^j^ ≥ 2.5	(PUFAs + MUFAs)/SFAs ≤ 1.2
Moderation components			
Refined grains	10	≤ 1.8 oz. equiv. per 1,000 kcal	≥ 4.3 oz. equiv. per 1,000 kcal
Sodium	10	≤ 1.1 grams per 1,000 kcal	≥ 2.0 grams per 1,000 kcal
Added sugars	10	< 6.5% of energy	≥ 26% of energy
Saturated fats	10	≤ 8% of energy	≥ 16% of energy

a Intakes between the minimum and maximum standards are scored proportionately.

b Includes 100% fruit juice.

c Includes all forms except juice.

d Includes beans, peas, and lentils.

e Includes all milk products, such as fluid milk, yogurt, and cheese, and fortified soy beverages.

f Includes seafood, nuts, seeds, soy products (other than beverages), and beans, peas, and lentils.

g Ratio of poly- and monounsaturated fatty acids (PUFAs and MUFAs) to saturated fatty acids (SFAs).

h PUFAs = polyunsaturated fatty acids.

i MUFAS = monounsaturated fatty acids.

j SFAs = saturated fatty acids.

### Data collection

Within the PLCO trial, baseline demographic and lifestyle data were gathered from participants using self-administered questionnaires (BQ). The key variables analyzed in our study encompassed age, sex, race, marriage, education level, smoking status, daily cigarette consumption, BMI at baseline, history of aspirin use, diabetes, hypertension, colorectal diverticulitis/diverticulosis, colorectal polyp and colorectal comorbidities (specifically Gardner’s syndrome, ulcerative colitis, Crohn’s disease, or familial polyposis), and family history of CRC. BMI was computed as weight (kg) divided by height squared (m^2^). Dietary intake information was collected using a validated 137-item Food Frequency Questionnaire (FFQ), termed the DHQ, which was administered 3 years post-enrollment in the PLCO trial. The DHQ evaluated portion sizes, frequencies, and types of foods and supplements consumed by participants over the preceding year. The validity of the DHQ was established through comparison with a 24-h dietary recall study (i.e., the Eating at America’s Table Study) (19). In this study, the DHQ demonstrated superior performance in assessing absolute nutrient intake compared to other commonly utilized FFQs, such as the Block and Willett questionnaires (19).

### Ascertainment of outcome events

In this study, CRC diagnosis was the primary outcome. CRC was defined according to the International Classification of Diseases for Oncology (ICD-O-2) codes: proximal colon cancer (C180–C185), distal colon cancer (C186–C187), and rectal cancer (C199–C209). Proximal colon cancer includes cancers of the cecum, appendix, ascending colon, hepatic flexure, transverse colon, and splenic flexure, while distal colon cancer includes cancers of the descending and sigmoid colon.

Participants received an annual self-report questionnaire to report any new CRC diagnoses, including diagnosis date and cancer type. If the questionnaire was not returned, follow-up was conducted via a second questionnaire or telephone. Medical records were reviewed as Supplementary material to confirm diagnoses. If participants died, family reports were collected, and death certificates, autopsy reports (if available), pathology slides, and other medical documents were examined to determine the underlying cause of death (20).

### Statistical analysis

**Figure 2:**
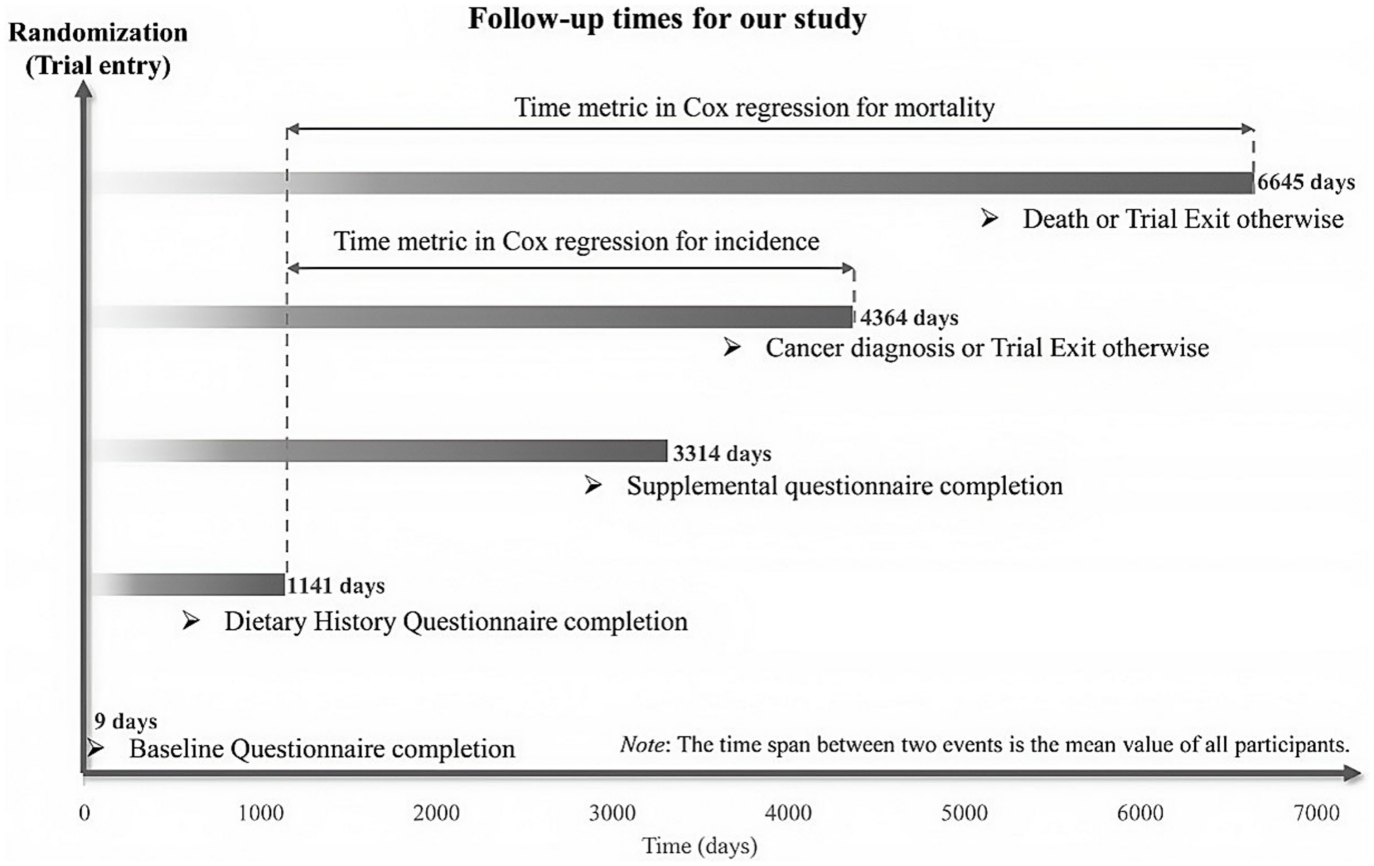
The timeline and follow-up scheme of our study.

In this analysis, covariates with less than 5% missing data were imputed using the mode for categorical variables and the median for continuous variables. Details of the missing data are shown in Supplementary Table 1. The primary statistical method used to examine the relationship between HEI-2020 scores and CRC incidence and mortality was the Cox proportional hazards regression model. Follow-up duration was defined as the time between DHQ completion and CRC diagnosis, CRC-related death, other causes of mortality, loss to follow-up, or study completion (with cancer data collected until December 31, 2009, and mortality data through 2018, as shown in Figure 2). Person-years were calculated based on follow-up duration. Participants were grouped into quartiles of HEI-2020 scores, with the first quartile serving as the reference group. Two multivariable Cox models were constructed. Model 1 was adjusted for age, sex, race, and marital status. Model 2 was further adjusted for hypertension, diabetes, smoking status, trial arm, daily cigarette consumption, alcohol drinking status, body mass index, aspirin use, family history of CRC, colorectal comorbidities, colorectal polyps, and diverticulitis/diverticulosis. For CRC-specific mortality, deaths from causes other than CRC were additionally considered as competing events. A Fine–Gray subdistribution hazard model was fitted to estimate subdistribution hazard ratios (SHRs) and 95% confidence intervals (CIs) for CRC-specific death, using the same covariate adjustment strategy as in the Cox models. CRC-specific death was defined as death among participants with confirmed incident CRC for whom CRC was recorded as the underlying cause of death. Participants who were alive, lost to follow-up, or reached the end of mortality follow-up without CRC-specific death were censored (21). The proportional hazards assumption for the Cox regression models was evaluated using Schoenfeld residuals. No substantial violation was observed in the main models. Specifically, for CRC incidence, the Schoenfeld residual test *p* value for HEI-2020 quartiles was 0.309 and the global test p value was 0.829; for CRC-specific mortality, the corresponding *p* values were 0.833 and 0.347, respectively. Restricted cubic spline analyses were performed using Cox proportional hazards models with HEI-2020 score as a continuous variable. Three knots were placed at the 10th, 50th, and 90th percentiles of the HEI-2020 distribution, corresponding to HEI-2020 scores of 53.39, 67.26, and 78.60, respectively. The median HEI-2020 score of 67.26 was used as the reference value. The spline models were adjusted for the same covariates as Model 2. Nonlinearity was assessed using the Wald test from the anova function in the rms package (22, 23). Subgroup analyses assessed the modification effects of key variables, including age, sex, race, marital status, diabetes history, smoking habits, history of colorectal comorbidities, BMI, aspirin use, and family history of CRC (as detailed in Supplementary Tables 3, 4). Interaction effects were evaluated by comparing models with and without interaction terms.

To further validate the results, sensitivity analyses were performed (2, 24): (1) excluding cases from the first two years of follow-up; (2) adjusting for pack-years of smoking instead of daily cigarette consumption (ranging from 0 to 20 cigarettes) for better statistical power; (3) excluding individuals with a history of diverticulitis/diverticulosis or colorectal comorbidities (such as ulcerative colitis, Crohn’s disease, Gardner’s syndrome, or familial polyposis); (4) excluding individuals with extreme BMI values (top and bottom 1%) (as detailed in Supplementary Tables 5, 6).

All statistical analyses were conducted using R software version 4.4.3, and a two-tailed *p*-value of < 0.05 was considered statistically significant.

## Results

### Participant baseline features

The study included 101,709 adults aged 55–74 years at baseline, stratified by Healthy Eating Index–2020 (HEI-2020) score quartiles to assess adherence to the 2020 Dietary Guidelines for Americans (DGA). Quartiles were defined as: Quartile 1 (≤60), Quartile 2 (61–67), Quartile 3 (68–74), Quartile 4 (>74). Demographically, compared with Q1, Q4 participants were more likely to be female (63.09% vs. 38.08%), non-white (8.59% vs. 6.63%), unmarried (23.27% vs. 22.07%), and hold postgraduate degrees (24.80% vs. 12.56%). Conversely, Q1 had a higher proportion of males (61.92% vs. 36.91%) and a higher proportion with ≤college education (73.35% vs. 54.70% in Q4). Regarding lifestyle factors, the proportion of current smokers was lower in Q4 than in Q1 (46.76% vs. 60.22%). Aspirin use was slightly more common in Q4 (47.41% vs. 46.79%). For clinical characteristics, the median daily energy intake was higher in Q4 (1137.72 ± 522.24 kcal vs. 910.70 ± 516.57 kcal in Q1), while the mean body mass index (BMI) was marginally lower (26.23 ± 4.48 kg/m^2^ vs. 27.91 ± 4.93 kg/m^2^) (as detailed in the Table 2).

**Table 2:** Baseline characteristics of study population according to overall HEI-2020.

Characteristics	Overall	Quartiles of overall HEI-2020			
		Quartile 1	Quartile 2	Quartile 3	Quartile 4
Number of participants	101,709	25,428	25,427	25,427	25,427
Age	62.40 ± 5.28	61.77 ± 5.20	62.37 ± 5.27	62.54 ± 5.26	62.93 ± 5.33
Sex					
Male	49,459 (48.63%)	15,746 (61.92%)	13,095 (51.50%)	11,234 (44.18%)	9,384 (36.91%)
Female	52,250 (51.37%)	9,682 (38.08%)	12,332 (48.50%)	14,193 (55.82%)	16,043 (63.09%)
Race					
White	94,023 (92.44%)	23,742 (93.37%)	23,620 (92.89%)	23,417 (92.10%)	23,244 (91.41%)
Non-white	7,686 (7.56%)	1,686 (6.63%)	1807 (7.11%)	2010 (7.90%)	2,183 (8.59%)
Education level					
College below	64,923 (63.83%)	18,629 (73.26%)	16,789 (66.03%)	15,602 (61.36%)	13,903 (54.68%)
College graduate	17,841 (17.54%)	3,604 (14.17%)	4,342 (17.08%)	4,677 (18.39%)	5,218 (20.52%)
Postgraduate	18,945 (18.63%)	3,195 (12.56%)	4,296 (16.90%)	5,148 (20.25%)	6,306 (24.80%)
Marriage					
Married	79,788 (78.45%)	19,817 (77.93%)	20,255 (79.66%)	20,207 (79.47%)	19,509 (76.73%)
Unmarried	21,921 (21.55%)	5,611 (22.07%)	5,172 (20.34%)	5,220 (20.53%)	5,918 (23.27%)
Diabetes history					
No	94,907 (93.31%)	23,807 (93.63%)	23,647 (93.00%)	23,644 (92.99%)	23,809 (93.64%)
Yes	6,802 (6.69%)	1,621 (6.37%)	1780 (7.00%)	1783 (7.01%)	1,618 (6.36%)
Aspirin use history					
No	53,927 (53.02%)	13,531 (53.21%)	13,498 (53.09%)	13,525 (53.19%)	13,373 (52.59%)
Yes	47,782 (46.98%)	11,897 (46.79%)	11,929 (46.91%)	11,902 (46.81%)	12,054 (47.41%)
Family history of colorectal cancer					
No	88,910 (87.42%)	22,127 (87.02%)	22,216 (87.37%)	22,236 (87.45%)	22,331 (87.82%)
Yes	10,306 (10.13%)	2,505 (9.85%)	2,570 (10.11%)	2,603 (10.24%)	2,628 (10.34%)
Possibly	2,493 (2.45%)	796 (3.13%)	641 (2.52%)	588 (2.31%)	468 (1.84%)
Diverticulitis/diverticulosis history					
No	94,886 (93.29%)	23,898 (93.98%)	23,735 (93.35%)	23,613 (92.87%)	23,640 (92.97%)
Yes	6,823 (6.71%)	1,530 (6.02%)	1,692 (6.65%)	1814 (7.13%)	1787 (7.03%)
Colorectal comorbidities history					
No	100,353 (98.67%)	25,072 (98.60%)	25,065 (98.58%)	25,085 (98.65%)	25,131 (98.84%)
Yes	1,356 (1.33%)	356 (1.40%)	362 (1.42%)	342 (1.35%)	296 (1.16%)
Colorectal polyp history					
No	94,944 (93.35%)	23,791 (93.56%)	23,744 (93.38%)	23,696 (93.19%)	23,713 (93.26%)
Yes	6,765 (6.65%)	1,637 (6.44%)	1,683 (6.62%)	1731 (6.81%)	1714 (6.74%)
Hypertension history					
No	68,678 (67.52%)	17,036 (67.00%)	16,724 (65.77%)	17,170 (67.53%)	17,748 (69.80%)
Yes	33,031 (32.48%)	8,392 (33.00%)	8,703 (34.23%)	8,257 (32.47%)	7,679 (30.20%)
Family history of cancer					
No	44,876 (44.12%)	11,361 (44.68%)	11,311 (44.48%)	11,125 (43.75%)	11,079 (43.57%)
Yes	56,833 (55.88%)	14,067 (55.32%)	14,116 (55.52%)	14,302 (56.25%)	14,348 (56.43%)
Arm					
Intervention	51,778 (50.91%)	12,828 (50.45%)	12,836 (50.48%)	12,989 (51.08%)	13,125 (51.62%)
Control	49,931 (49.09%)	12,600 (49.55%)	12,591 (49.52%)	12,438 (48.92%)	12,302 (48.38%)
Smoking status					
No	48,562 (47.75%)	10,116 (39.78%)	12,056 (47.41%)	12,852 (50.54%)	13,538 (53.24%)
Current/former	53,147 (52.25%)	15,312 (60.22%)	13,371 (52.59%)	12,575 (49.46%)	11,889 (46.76%)
Body mass index at baseline (kg/m^2^)	27.22 ± 4.79	27.91 ± 4.93	27.63 ± 4.82	27.13 ± 4.73	26.23 ± 4.48
Weight fluctuation ^a^	2.88 ± 0.76	2.99 ± 0.75	2.95 ± 0.75	2.87 ± 0.75	2.72 ± 0.74
Smoking pack-years	17.65 ± 26.59	24.78 ± 31.73	18.25 ± 26.95	15.10 ± 23.76	12.47 ± 21.10
Daily cigarette consumption					
0	48,666 (47.85%)	10,146 (39.90%)	12,083 (47.52%)	12,880 (50.65%)	13,557 (53.32%)
1–20	33,203 (32.65%)	8,413 (33.09%)	8,235 (32.39%)	8,247 (32.43%)	8,308 (32.67%)
>20	19,840 (19.51%)	6,869 (27.01%)	5,109 (20.09%)	4,300 (16.91%)	3,562 (14.01%)
Energy	1006.24 ± 519.00	910.70 ± 516.57	960.00 ± 502.17	1016.57 ± 507.02	1137.72 ± 522.24
Drinker					
No	27,741 (27.27%)	7,565 (29.75%)	6,907 (27.16%)	6,671 (26.24%)	6,598 (25.95%)
Yes	73,968 (72.73%)	17,863 (70.25%)	18,520 (72.84%)	18,756 (73.76%)	18,829 (74.05%)

Descriptive statistics are presented as (mean ± standard deviation) and number (percentage) for continuous and categorical.

a Weight fluctuation was defined as the participant’s baseline weight minus weight at age 20.

### Association between CRC incidence and HEI-2020

**Figure 3:**
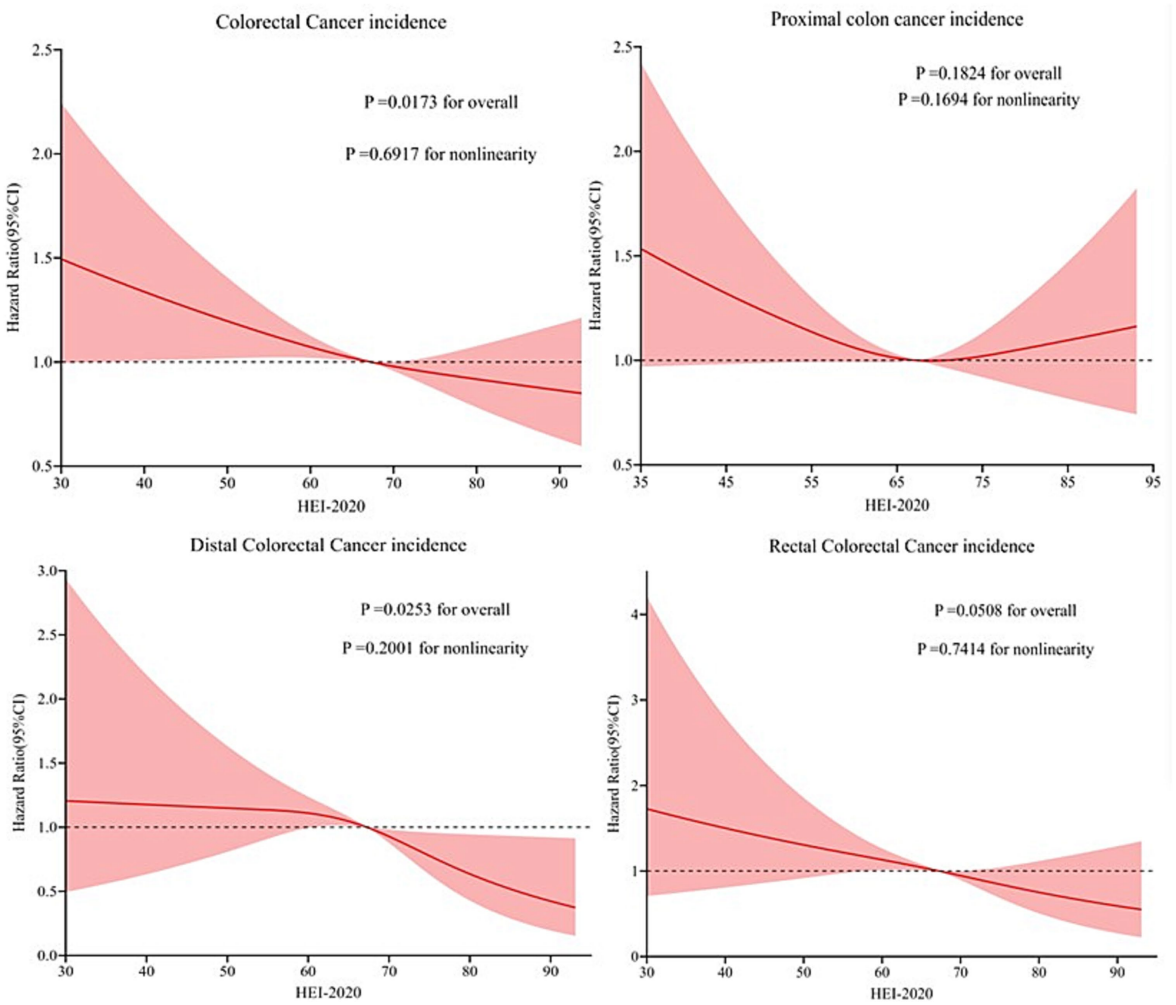
The RCS model was used to analyze the association between HEI-2020 and the incidence of colorectal cancer, proximal colon cancer, distal colorectal cancer, and rectal cancer. Hazard ratio was adjusted for age (years), sex (male, female), race (white and non-white), marital status (married, unmarried), history of hypertension (yes, no), history of diabetes (yes, no), smoking status (never, currently/ever), trial arm (intervention, control), number of cigarettes smoked (0, 1–20, > 20 cigarettes/day), history of alcohol consumption (yes, no), body mass index (kg/m^2^), aspirin use (yes, no), family history of colorectal cancer (yes, no), history of colorectal comorbidities (yes, no), history of colorectal polyps (yes, no), and history of colorectal diverticulitis/diverticulosis (yes, no).

During a median follow-up of 8.83 years, 1,100 incident CRC cases were identified among 101,709 participants, corresponding to 896,109.1 person-years of observation; these cases included 648 proximal colon cancers, 226 distal colon cancers, and 204 rectal cancers. In the fully adjusted model, participants in the highest HEI-2020 quartile had a lower point estimate for overall CRC incidence than those in the lowest quartile, although the Q4 versus Q1 comparison did not reach statistical significance (HR = 0.86; 95% CI: 0.73–1.02). Nevertheless, a statistically significant decreasing trend in CRC incidence risk was observed across HEI-2020 quartiles (P-trend = 0.021; as detailed in Table 3). Anatomical subsite analysis showed no significant association between HEI-2020 scores and proximal colon cancer risk (Q4 vs. Q1: HR = 0.97; 95% CI: 0.78–1.20; P-trend = 0.561). Higher HEI-2020 adherence was associated with a lower risk of distal colon cancer (Q4 vs. Q1: HR = 0.65; 95% CI: 0.43–0.98; P-trend = 0.013) and showed a borderline lower point estimate for rectal cancer (Q4 vs. Q1: HR = 0.66; 95% CI: 0.44–1.00; P-trend = 0.028; as detailed in Table 3). Moreover, restricted cubic spline analyses suggested an overall inverse association between HEI-2020 scores and overall CRC incidence, without evidence of nonlinearity (P for overall association = 0.017; P for nonlinearity = 0.692; as detailed in Figure 3). Similar patterns were observed in the subsite-specific RCS analyses for distal colon cancer and rectal cancer, with no significant nonlinearity observed (as detailed in Figure 3).

**Table 3:** Hazard ratios of the association between HEI-2020 and CRC incidence.

Quartiles of HEI-2020	Cases	Person-years	Incidence rate per 10,000 person-years (95% confidence interval)	Hazard ratio (95% confidence interval) by HEI-2020		
				Unadjusted	Model 1^a^	Model 2^b^
Colorectal cancer						
Quartile 1	302	220,176.0	13.72 (12.26,15.35)	1.000 (reference)	1.000 (reference)	1.000 (reference)
Quartile 2	297	223,454.7	13.29 (11.86,14.89)	0.97 (0.83, 1.14)	0.96 (0.81, 1.12)	0.97 (0.82, 1.14)
Quartile 3	241	225,703.3	10.68 (9.41,12.11)	0.78(0.66, 0.92)	0.77 (0.65, 0.92)	0.79 (0.67, 0.94)
Quartile 4	260	226,775.1	11.47 (10.15,12.95)	0.84 (0.71, 0.99)	0.82 (0.69, 0.97)	0.86 (0.73, 1.02)
p for trend				0.006	0.004	0.021
Proximal colon cancer						
Quartile 1	170	220,176.0	7.23 (6.70, 7.81)	1.000 (reference)	1.000 (reference)	1.000 (reference)
Quartile 2	158	223,454.7	7.72 (6.64, 8.97)	0.92 (0.74, 1.14)	0.88 (0.71, 1.10)	0.88 (0.71, 1.10)
Quartile 3	145	225,703.3	6.42 (5.46, 7.56)	0.83 (0.67, 1.04)	0.79 (0.64, 0.99)	0.80 (0.64, 1.00)
Quartile 4	175	226,775.1	7.72 (6.66, 8.95)	1.00 (0.81, 1.23)	0.93 (0.75, 1.15)	0.97 (0.78, 1.20)
p for trend				0.761	0.355	0.561
Distal colon cancer						
Quartile 1	64	220,176.0	2.91 (2.28, 3.71)	1.000 (reference)	1.000 (reference)	1.000 (reference)
Quartile 2	75	223,454.7	3.36 (2.68, 4.21)	1.16 (0.83, 1.62)	1.15 (0.83, 1.61)	1.18 (0.84, 1.65)
Quartile 3	47	225,703.3	2.08 (1.57, 2.77)	0.72 (0.49, 1.05)	0.72 (0.49, 1.06)	0.75 (0.51, 1.10)
Quartile 4	40	226,775.1	1.76 (1.30, 2.40)	0.61 (0.41, 0.90)	0.61 (0.41, 0.92)	0.65 (0.43, 0.98)
p for trend				0.003	0.004	0.013
Rectal cancer						
Quartile 1	62	220,176.0	2.82 (2.16, 3.61)	1.000 (reference)	1.000 (reference)	1.000 (reference)
Quartile 2	59	223,454.7	2.64 (2.01, 3.41)	0.90 (0.63, 1.28)	0.94 (0.66, 1.35)	0.98 (0.68, 1.40)
Quartile 3	44	225,703.3	1.95 (1.42, 2.62)	0.65 (0.44, 0.95)	0.70 (0.47, 1.03)	0.74 (0.50, 1.10)
Quartile 4	39	226,775.1	1.72 (1.22, 2.35)	0.55 (0.37, 0.82)	0.61 (0.40, 0.91)	0.66 (0.44, 1.00)
p for trend				0.001	0.007	0.028

a Model 1 was adjusted for age (continuous), sex (male, female), race (white, non-white), and marital status (married, unmarried).

b Model 2 was adjusted for Model 1 plus BMI at baseline (continuous), trial arm (intervention, control), smoking status (never, current or former), daily cigarette consumption (0, 1–20, >20), aspirin use (no, yes), family history of colorectal cancer (no, yes, possibly), history of hypertension (no, yes), history of diabetes (no, yes), history of colorectal diverticulitis/diverticulosis (no, yes), history of colorectal comorbidities (no, yes), history of colorectal polyps (no, yes), and history of alcohol consumption (no, yes).

### Association between CRC mortality and HEI-2020

**Figure 4:**
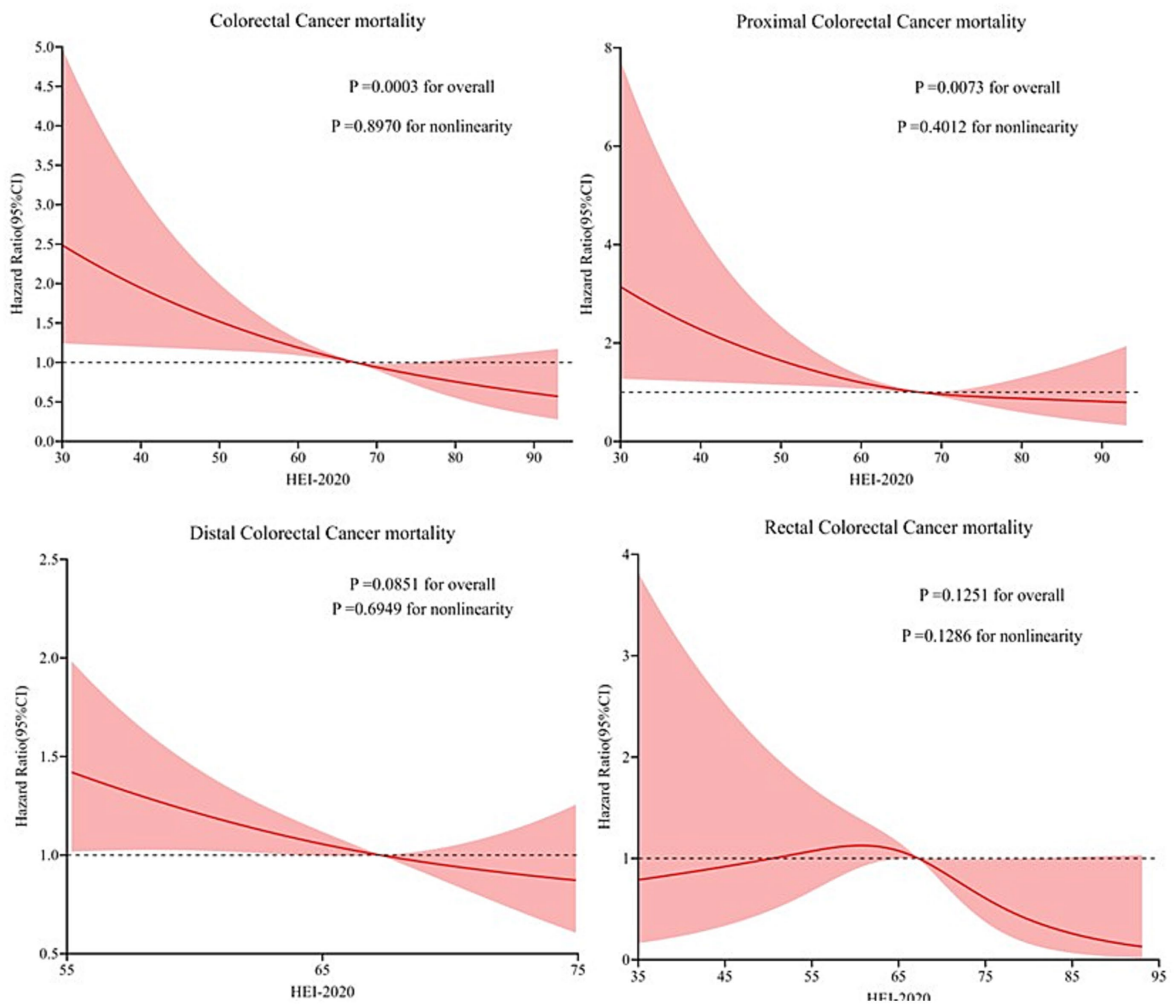
The RCS model was used to analyze the association between HEI-2020 and the mortality of colorectal cancer, proximal colon cancer, distal colorectal cancer, and rectal cancer. Hazard ratio was adjusted for age (years), sex (male, female), race (white and non-white), marital status (married, unmarried), history of hypertension (yes, no), history of diabetes (yes, no), smoking status (never, currently/ever), trial arm (intervention, control), number of cigarettes smoked (0, 1–20, > 20 cigarettes/day), history of alcohol consumption (yes, no), body mass index (kg/m^2^), aspirin use (yes, no), family history of colorectal cancer (yes, no), history of colorectal comorbidities (yes, no), history of colorectal polyps (yes, no), and history of colorectal diverticulitis/diverticulosis (yes, no).

Over 1,532,681 person-years of follow-up, 314 CRC-specific deaths were recorded, including 184 proximal colon cancer deaths, 71 distal colon cancer deaths, and 54 rectal cancer deaths. In the fully adjusted model, participants in the highest HEI-2020 quartile had a 39% lower overall CRC mortality risk versus those in the lowest quartile (HR = 0.61; 95% CI: 0.45–0.85), with a statistically significant linear trend (P-trend = 0.001; as detailed in Table 4). When deaths from causes other than CRC were treated as competing events, the association remained consistent. In the fully adjusted Fine–Gray model, participants in the highest HEI-2020 quartile had a lower subdistribution hazard of CRC-specific death than those in the lowest quartile (SHR = 0.64; 95% CI: 0.46–0.88; *p* = 0.007), with a significant trend across quartiles (P for trend = 0.004; as detailed in Supplementary Table 2). Anatomical subsite analysis showed a decreasing trend in proximal colon cancer mortality across HEI-2020 quartiles, although the Q4 versus Q1 comparison did not reach statistical significance (HR = 0.67; 95% CI: 0.45–1.01; P-trend = 0.041). No statistically significant trends were observed for distal colon cancer mortality (HR = 0.66; 95% CI: 0.33–1.32; P-trend = 0.109) or rectal cancer mortality (HR = 0.36; 95% CI: 0.14–0.93; P-trend = 0.066; as detailed in Table 4). Moreover, restricted cubic spline analyses suggested an overall inverse association between HEI-2020 scores and CRC-specific mortality, without evidence of nonlinearity (P for overall association <0.001; P for nonlinearity = 0.897; as detailed in Figure 4). Similar patterns were observed in the subsite-specific RCS analyses for proximal colon cancer mortality, with no significant nonlinearity observed (as detailed in Figure 4).

**Table 4:** Hazard ratios of the association between HEI-2020 and CRC mortality.

Quartiles of HEI-2020	Cases	Person-years	Incidence rate per 10,000 person-years (95% confidence interval)	Hazard ratio (95% confidence interval) by HEI-2020		
				Unadjusted	Model 1^a^	Model 2^b^
Colorectal cancer						
Quartile 1	105	371,196.3	2.83 (2.34, 3.42)	1.000 (reference)	1.000 (reference)	1.000 (reference)
Quartile 2	77	379,914.9	2.03 (1.62, 2.53)	0.72 (0.54, 0.97)	0.71 (0.53, 0.96)	0.73 (0.54, 0.98)
Quartile 3	68	386,608.6	1.76 (1.39, 2.23)	0.63 (0.46, 0.85)	0.62 (0.46, 0.85)	0.65 (0.48, 0.89)
Quartile 4	64	394,961.2	1.62 (1.27, 2.07)	0.58 (0.43, 0.79)	0.56 (0.41, 0.77)	0.61 (0.45, 0.85)
p for trend				<0.001	<0.001	0.001
Proximal colon cancer						
Quartile 1	61	371,196.3	1.64 (1.28, 2.11)	1.000 (reference)	1.000 (reference)	1.000 (reference)
Quartile 2	39	379,914.9	1.03 (0.75, 1.40)	0.63 (0.42, 0.94)	0.60 (0.40, 0.91)	0.62 (0.41, 0.93)
Quartile 3	40	386,608.6	1.03 (0.76, 1.41)	0.64 (0.43, 0.95)	0.60 (0.40, 0.90)	0.63 (0.42, 0.94)
Quartile 4	44	394,961.2	1.11 (0.83, 1.50)	0.69 (0.47, 1.02)	0.63 (0.42, 0.94)	0.67 (0.45, 1.01)
p for trend				0.046	0.017	0.041
Distal colon cancer						
Quartile 1	24	371,196.3	0.65 (0.43, 0.96)	1.000 (reference)	1.000 (reference)	1.000 (reference)
Quartile 2	22	379,914.9	0.58 (0.38, 0.88)	0.90 (0.51, 1.61)	0.93 (0.52, 1.66)	0.96 (0.54, 1.73)
Quartile 3	12	386,608.6	0.31 (0.18, 0.54)	0.49 (0.24, 0.97)	0.52 (0.26, 1.05)	0.56 (0.28, 1.12)
Quartile 4	13	394,961.2	0.33 (0.19, 0.56)	0.52 (0.26, 1.02)	0.57 (0.29, 1.13)	0.66 (0.33, 1.32)
p for trend				0.018	0.042	0.109
Rectal cancer						
Quartile 1	18	371,196.3	0.48 (0.31, 0.77)	1.000 (reference)	1.000 (reference)	1.000 (reference)
Quartile 2	15	379,914.9	0.39 (0.24, 0.65)	0.81 (0.41, 1.62)	0.83 (0.42, 1.65)	0.87 (0.44, 1.74)
Quartile 3	15	386,608.6	0.39 (0.24, 0.64)	0.80 (0.40, 1.59)	0.83 (0.41, 1.66)	0.90 (0.45, 1.81)
Quartile 4	6	394,961.2	0.15 (0.07, 0.33)	0.31 (0.12, 0.79)	0.32 (0.13, 0.82)	0.36 (0.14, 0.93)
p for trend				0.021	0.029	0.066

a Model 1 was adjusted for age (continuous), sex (male, female), race (white, non-white), and marital status (married, unmarried).

b Model 2 was adjusted for Model 1 plus BMI at baseline (continuous), trial arm (intervention, control), smoking status (never, current or former), daily cigarette consumption (0, 1–20, >20), aspirin use (no, yes), family history of colorectal cancer (no, yes, possibly), history of hypertension (no, yes), history of diabetes (no, yes), history of colorectal diverticulitis/diverticulosis (no, yes), history of colorectal comorbidities (no, yes), history of colorectal polyps (no, yes), and history of alcohol consumption (no, yes).

### Additional analyses

In the analysis of the association between the HEI-2020 score and CRC incidence and CRC-specific mortality, no statistically significant interaction was observed across any predefined subgroups (as detailed in Supplementary Tables 3, 4). For CRC incidence, the inverse association between HEI-2020 and CRC risk appeared more evident among participants with BMI ≤ 30 kg/m^2^. In this subgroup, participants in Quartile 4 had a lower CRC risk than those in Quartile 1 (HR = 0.80; 95% CI: 0.66–0.97; P for trend = 0.021), whereas no similar pattern was observed among participants with BMI > 30 kg/m^2^. For CRC-specific mortality, inverse associations were also more apparent in several subgroups, including participants aged >65 years, men, White participants, married participants, current/former smokers, and those with BMI ≤ 30 kg/m^2^. Because the formal interaction tests were not statistically significant, these subgroup patterns should be interpreted cautiously and considered exploratory.

Sensitivity analyses further supported the robustness of the primary findings (as detailed in Supplementary Tables 5, 6). The inverse association between the HEI-2020 score and CRC outcomes remained generally consistent after excluding cases occurring within the first 2 years of follow-up, removing extreme BMI values (top and bottom 1%), excluding participants with diverticulitis/diverticulosis or other colorectal-related comorbidities, and adjusting for smoking using pack-years instead of categorical smoking status. These results collectively support the stability of the observed associations.

## Discussion

Our findings suggest that higher HEI-2020 scores were associated with a lower point estimate for CRC incidence, although the highest versus lowest quartile comparison did not reach statistical significance. However, a significant decreasing trend in CRC incidence risk was observed across HEI-2020 quartiles. In contrast, higher HEI-2020 scores were significantly associated with lower CRC-specific mortality, and this association remained consistent after accounting for deaths from causes other than CRC as competing events. RCS analyses suggested overall inverse associations of HEI-2020 with CRC incidence and CRC-specific mortality, without evidence of nonlinearity. Sensitivity analyses further supported the stability of these findings.

Several components of the HEI score are biologically linked to pathways relevant to colorectal carcinogenesis. Plant-derived foods, including fruits, vegetables, legumes, and whole grains, provide phenolic acids, flavonoids, polyphenols, carotenoids, and folate (25–27). These compounds influence multiple molecular processes involved in early and late tumor development. Polyphenols have been shown to attenuate intestinal inflammation by reducing TNF–TNFR signaling, inhibiting NF-κB activation, and lowering pro-inflammatory cytokines such as TNF-*α*, IL-1β, IL-6, and IL-8. They also modulate MAPK and PI3K/Akt pathways and shift the balance of Bcl-2, Bcl-xL, and Bax toward apoptosis (28). Carotenoids contribute to the reduction of oxidative stress, support DNA repair, and limit the formation of aberrant crypt foci (25), while glucosinolates and isothiocyanates from cruciferous vegetables induce G1 or G2/M cell-cycle arrest and suppress invasive properties of colorectal cancer cells (27). Folate plays an additional role in maintaining genomic stability through its involvement in nucleotide synthesis and DNA methylation (29).

Whole grains and legumes are major sources of fermentable dietary fiber, which is metabolized by colonic microbiota into short-chain fatty acids (SCFAs), particularly butyrate (30, 31). SCFAs inhibit histone deacetylase activity, induce G1 arrest, and promote apoptosis through G-protein–coupled receptor signaling and regulation of Bcl-2 family proteins. They also down-regulate VEGF and ERK/MAPK signaling, contributing to reduced proliferative and angiogenic activity (32, 33). By supporting a more diverse microbiota and enhancing epithelial barrier integrity, high-fiber diets counteract microbial and inflammatory drivers of colorectal tumorigenesis (34).

Dairy products, primarily through their calcium content, may mitigate colorectal cancer risk by binding secondary bile acids and free fatty acids in the intestinal lumen, reducing their cytotoxicity and proliferative stimulation of colonocytes. Calcium also influences epithelial differentiation and apoptosis via calcium-sensing receptor activation (35, 36). Fermented dairy products may further support mucosal repair and reduce preneoplastic lesion formation (37).

In contrast, dietary components that HEI encourages limiting—added sugars, refined grains, and saturated fat—are associated with metabolic and inflammatory pathways that promote carcinogenesis. High glycemic diets increase post-prandial glucose and insulin levels, leading to elevated circulating IGF-1, which activates PI3K/Akt/mTOR and Ras/Raf/MEK/ERK pathways, suppresses apoptosis, and enhances expression of oncogenes such as MYC and KRAS, as well as angiogenic mediators including VEGF (38). Excess saturated fat intake promotes bile acid synthesis and TLR4-mediated inflammatory signaling, whereas omega-3 fatty acids exert an opposing anti-inflammatory effect and support immunoregulatory macrophage phenotypes (39–41). High sodium intake may contribute to mucosal inflammation and dysbiosis, although direct evidence for colorectal carcinogenesis remains limited (42–44).

Together, these mechanisms provide a biological framework explaining why diets characterized by higher HEI scores—rich in plant-based foods, fiber, and calcium, and lower in sugars, refined carbohydrates, and saturated fats—are associated with reduced colorectal cancer risk.

The subgroup findings suggested that the inverse association between HEI-2020 and CRC incidence may be more apparent among participants with BMI ≤ 30 kg/m^2^, although the interaction by BMI was not statistically significant. This pattern should therefore be interpreted cautiously. Obesity has been linked to colorectal carcinogenesis through multiple biological pathways, including chronic low-grade inflammation, insulin resistance, altered adipokine and hormone signaling, gut microbiota dysregulation, and bile acid metabolism. These obesity-related metabolic and inflammatory disturbances may partly attenuate the potential benefits of a high-quality diet. Further studies are needed to clarify whether obesity status modifies the association between diet quality and CRC risk (45).

Our study has several strengths. Firstly, our study data are derived from a large prospective cohort comprising over 100,000 participants with diverse occupational backgrounds across 10 screening centers in the United States, ensuring broad representativeness of the study population (18). The long-term follow-up ensured the reliability of the study findings. Secondly, the prospective design of the PLCO study effectively reduced the possibility of reverse causation due to subclinical pathological conditions leading to dietary changes, enhancing the credibility of the observed associations (46). The study rigorously controlled for selection bias by ensuring comparable proportions of CRC diagnoses between excluded and included populations, further strengthening internal validity. After comprehensive adjustment for multiple potential confounders, the reliability of the study results was reinforced. Most importantly, this study represents the first systematic investigation of the relationship between HEI-2020 and the incidence and mortality of CRC, providing crucial new evidence in this field. According to the study findings, a dietary pattern with a high HEI-2020 score was associated with a significant decreasing trend in CRC incidence risk and a lower risk of CRC-specific mortality, with generally consistent findings in sensitivity analyses.

Despite its strengths, several limitations should be acknowledged. First, dietary intake was assessed only once using the DHQ. Changes in dietary habits during the long follow-up period could not be captured, which may have introduced exposure misclassification and regression dilution bias and may have attenuated the observed associations. In addition, dietary intake was self-reported and therefore subject to measurement error and recall bias. Dietary measurement error is a well-recognized challenge in nutritional cohort studies and may reduce statistical power or bias diet–disease associations toward the null (47).

Second, although we adjusted for a range of demographic, lifestyle, and clinical factors, residual confounding cannot be fully excluded. Some potentially relevant factors, including physical activity, socioeconomic status, healthcare access, and actual CRC screening frequency, were not fully accounted for in the present analysis. Participants with higher HEI-2020 scores may also have been more likely to engage in other health-promoting behaviors or preventive healthcare practices, raising the possibility of a healthy user effect (48).

Third, due to the lack of blood or urine biomarkers, this study could not further validate the HEI-2020 score or explore the underlying biological mechanisms in greater depth. Finally, the PLCO cohort mainly included middle-aged and older U. S. adults and was predominantly composed of White participants; therefore, the generalizability of our findings to younger populations, other racial or ethnic groups, and non-U.S. populations may be limited.

Future studies with repeated dietary assessments, objective nutritional biomarkers, and more detailed information on lifestyle factors, socioeconomic status, healthcare access, and CRC screening behaviors are needed to further clarify the association between diet quality and CRC outcomes.

## Conclusion

In summary, greater adherence to the dietary pattern reflected by the HEI-2020 was associated with a significant decreasing trend in CRC incidence risk and a lower risk of CRC-specific mortality among U.S. adults. These findings provide prospective evidence supporting the potential relevance of overall diet quality in CRC prevention, while further studies are needed to confirm these associations in other populations.

## Data Availability

The data analyzed in this study are subject to NIH/NCI data access policies and are available from the PLCO Cancer Screening Trial/NCI Cancer Data Access System under approved access. The analytic results supporting the conclusions of this article are included in the article and Supplementary material. Further inquiries can be directed to the corresponding authors.

## References

[1] Siegel RL Kratzer TB Giaquinto AN Sung H Jemal A. Cancer statistics, 2025. CA Cancer J Clin. (2025) 75:10–45. doi: 10.3322/caac.21871, 39817679 PMC11745215

[2] Hossain MS Karuniawati H Jairoun AA Urbi Z Ooi DJ John A . Colorectal Cancer: a review of carcinogenesis, global epidemiology, current challenges, risk factors, preventive and treatment strategies. Cancers. (2022) 14:1732. doi: 10.3390/cancers14071732, 35406504 PMC8996939

[3] Papier K Bradbury KE Balkwill A Barnes I Smith-Byrne K Gunter MJ . Diet-wide analyses for risk of colorectal cancer: prospective study of 12,251 incident cases among 542,778 women in the UK. Nat Commun. (2025) 16:375. doi: 10.1038/s41467-024-55219-5, 39779669 PMC11711514

[4] Keum N Giovannucci E. Global burden of colorectal cancer: emerging trends, risk factors and prevention strategies. Nat Rev Gastroenterol Hepatol. (2019) 16:713–32. doi: 10.1038/s41575-019-0189-8, 31455888

[5] Bradbury KE Murphy N Key TJ. Diet and colorectal cancer in UK biobank: a prospective study. Int J Epidemiol. (2020) 49:246–58. doi: 10.1093/ije/dyz064, 30993317 PMC7124508

[6] Schwingshackl L Schwedhelm C Hoffmann G Knüppel S Laure Preterre A Iqbal K . Food groups and risk of colorectal cancer. Int J Cancer. (2018) 142:1748–58. doi: 10.1002/ijc.31198, 29210053

[7] Caini S Chioccioli S Pastore E Fontana M Tortora K Caderni G . Fish consumption and colorectal Cancer risk: Meta-analysis of prospective epidemiological studies and review of evidence from animal studies. Cancers (Basel). (2022) 14:640. doi: 10.3390/cancers14030640, 35158907 PMC8833371

[8] Hur J Otegbeye E Joh HK Nimptsch K Ng K Ogino S . Sugar-sweetened beverage intake in adulthood and adolescence and risk of early-onset colorectal cancer among women. Gut. (2021) 70:2330–6. doi: 10.1136/gutjnl-2020-323450, 33958435 PMC8571123

[9] Shams-White MM Pannucci TE Lerman JL Herrick KA Zimmer M Meyers Mathieu K . Healthy eating Index-2020: review and update process to reflect the dietary guidelines for Americans,2020-2025. J Acad Nutr Diet. (2023) 123:1280–8. doi: 10.1016/j.jand.2023.05.015, 37201748 PMC10524328

[10] Phillips JA. Dietary guidelines for Americans, 2020-2025. Workplace Health Saf. (2021) 69:395. doi: 10.1177/21650799211026980, 34279148

[11] Morze J Danielewicz A Hoffmann G Schwingshackl L. Diet quality as assessed by the healthy eating index, alternate healthy eating index, dietary approaches to stop hypertension score, and health outcomes: a second update of a systematic review and Meta-analysis of cohort studies. J Acad Nutr Diet. (2020) 120:1998–2031.e15. doi: 10.1016/j.jand.2020.08.076, 33067162

[12] Hao X Li D. The healthy eating Index-2015 and all-cause/cause-specific mortality: a systematic review and dose-response Meta-analysis. Adv Nutr. (2024) 15:100166. doi: 10.1016/j.advnut.2023.100166, 38461130 PMC10980904

[13] Zhang Y Lu C Li X Fan Y Li J Liu Y . Healthy eating Index-2015 and predicted 10-year cardiovascular disease risk, as well as heart age. Front Nutr. (2022) 9:888966. doi: 10.3389/fnut.2022.888966, 35903444 PMC9315384

[14] Kord-Varkaneh H Salehi-Sahlabadi A Zarezade M Rahmani J Tan SC Hekmatdoost A . Association between healthy eating Index-2015 and breast cancer risk: a case-control study. Asian Pac J Cancer Prev. (2020) 21:1363–7. doi: 10.31557/APJCP.2020.21.5.1363, 32458645 PMC7541885

[15] Edefonti V Di Maso M Tomaino L Parpinel M Garavello W Serraino D . Diet quality as measured by the healthy eating index 2015 and oral and pharyngeal cancer risk. J Acad Nutr Diet. (2022) 122:1677–1687.e5. doi: 10.1016/j.jand.2021.04.020, 34127426

[16] Prorok PC Andriole GL Bresalier RS Buys SS Chia D David Crawford E . Design of the Prostate, lung, colorectal and ovarian (PLCO) cancer screening trial. Control Clin Trials. (2000) 21:273S–309S. doi: 10.1016/s0197-2456(00)00098-2, 11189684

[17] Zhu CS Pinsky PF Kramer BS Prorok PC Purdue MP Berg CD . The prostate, lung, colorectal, and ovarian cancer screening trial and its associated research resource. J Natl Cancer Inst. (2013) 105:1684–93. doi: 10.1093/jnci/djt281, 24115361 PMC3888207

[18] Gohagan JK Prorok PC Hayes RB Kramer BS. The prostate, lung, colorectal and ovarian (PLCO) cancer screening trial of the National Cancer Institute: history, organization, and status. Control Clin Trials. (2000) 21:251S–72S. doi: 10.1016/s0197-2456(00)00097-0, 11189683

[19] Subar AF Thompson FE Kipnis V Midthune D Hurwitz P McNutt S . Comparative validation of the block, Willett, and National Cancer Institute food frequency questionnaires: the eating at America’s table study. Am J Epidemiol. (2001) 154:1089–99. doi: 10.1093/aje/154.12.1089, 11744511

[20] Gohagan JK Prorok PC Greenwald P Kramer BS. The PLCO cancer screening trial: background, goals, organization, operations, results. Rev Recent Clin Trials. (2015) 10:173–80. doi: 10.2174/1574887110666150730123004, 26238115

[21] Austin PC Lee DS Fine JP. Introduction to the analysis of survival data in the presence of competing risks. Circulation. (2016) 133:601–9. doi: 10.1161/CIRCULATIONAHA.115.017719, 26858290 PMC4741409

[22] Austin PC Fang J Lee DS. Using fractional polynomials and restricted cubic splines to model non-proportional hazards or time-varying covariate effects in the cox regression model. Stat Med. (2022) 41:612–24. doi: 10.1002/sim.9259, 34806210 PMC9299077

[23] Desquilbet L Mariotti F. Dose-response analyses using restricted cubic spline functions in public health research. Stat Med. (2010) 29:1037–57. doi: 10.1002/sim.3841, 20087875

[24] Johnson CM Wei C Ensor JE Smolenski DJ Amos CI Levin B . Meta-analyses of colorectal cancer risk factors. Cancer Causes Control. (2013) 24:1207–22. doi: 10.1007/s10552-013-0201-5, 23563998 PMC4161278

[25] Song M Garrett WS Chan AT. Nutrients, foods, and colorectal cancer prevention. Gastroenterology. (2015) 148:1244–1260.e16. doi: 10.1053/j.gastro.2014.12.035, 25575572 PMC4409470

[26] Alzate-Yepes T Pérez-Palacio L Martínez E Osorio M. Mechanisms of action of fruit and vegetable phytochemicals in colorectal Cancer prevention. Molecules. (2023) 28:4322. doi: 10.3390/molecules28114322, 37298797 PMC10254396

[27] Ren HG Luu HN Liu Y Wang DW Guo X. High intake of cruciferous vegetables reduces the risk of gastrointestinal cancers: results from observational studies. Crit Rev Food Sci Nutr. (2024) 64:8493–9. doi: 10.1080/10408398.2023.2271070, 38051036

[28] Bracci L Fabbri A Del Cornò M Conti L. Dietary polyphenols: promising adjuvants for colorectal Cancer therapies. Cancers. (2021) 13:4499. doi: 10.3390/cancers13184499, 34572726 PMC8465098

[29] Duthie SJ. Folate and cancer: how DNA damage, repair and methylation impact on colon carcinogenesis. J Inherit Metab Dis. (2011) 34:101–9. doi: 10.1007/s10545-010-9128-0, 20544289

[30] Gaesser GA. Whole grains, refined grains, and Cancer risk: a systematic review of Meta-analyses of observational studies. Nutrients. (2020) 12:3756. doi: 10.3390/nu12123756, 33297391 PMC7762239

[31] Zalila-Kolsi I Dhieb D Osman HA Mekideche H. The gut microbiota and colorectal Cancer: understanding the link and exploring therapeutic interventions. Biology. (2025) 14:251. doi: 10.3390/biology14030251, 40136508 PMC11939563

[32] He Y Peng K Tan J Hao Y Zhang S Gao C . Short-chain fatty acids and colorectal Cancer: a systematic review and integrative Bayesian Meta-analysis of microbiome-metabolome interactions and intervention efficacy. Nutrients. (2025) 17:3552. doi: 10.3390/nu17223552, 41305603 PMC12655149

[33] Liu P Wang Y Yang G Zhang Q Meng L Xin Y . The role of short-chain fatty acids in intestinal barrier function, inflammation, oxidative stress, and colonic carcinogenesis. Pharmacol Res. (2021) 165:105420. doi: 10.1016/j.phrs.2021.105420, 33434620

[34] Holscher HD. Dietary fiber and prebiotics and the gastrointestinal microbiota. Gut Microbes. (2017) 8:172–84. doi: 10.1080/19490976.2017.1290756, 28165863 PMC5390821

[35] Barrubés L Babio N Becerra-Tomás N Rosique-Esteban N Salas-Salvadó J. Association between dairy product consumption and colorectal Cancer risk in adults: a systematic review and Meta-analysis of epidemiologic studies. Adv Nutr. (2019) 10:S190–211. doi: 10.1093/advances/nmy114, 31089733 PMC6518136

[36] Norat T Riboli E. Dairy products and colorectal cancer. A review of possible mechanisms and epidemiological evidence. Eur J Clin Nutr. (2003) 57:1–17. doi: 10.1038/sj.ejcn.1601522, 12548291

[37] Illikoud N Mantel M Rolli-Derkinderen M Gagnaire V Jan G. Dairy starters and fermented dairy products modulate gut mucosal immunity. Immunol Lett. (2022) 251-252:91–102. doi: 10.1016/j.imlet.2022.11.002, 36334759

[38] Kasprzak A. Insulin-like growth factor 1 (IGF-1) signaling in glucose metabolism in colorectal Cancer. Int J Mol Sci. (2021) 22:6434. doi: 10.3390/ijms22126434, 34208601 PMC8234711

[39] Ocvirk S O’Keefe SJD. Dietary fat, bile acid metabolism and colorectal cancer. Semin Cancer Biol. (2021) 73:347–55. doi: 10.1016/j.semcancer.2020.10.003, 33069873

[40] Aglago EK Huybrechts I Murphy N Casagrande C Nicolas G Pischon T . Consumption of fish and long-chain n-3 polyunsaturated fatty acids is associated with reduced risk of colorectal Cancer in a large European cohort. Clin Gastroenterol Hepatol. (2020) 18:654–666.e6. doi: 10.1016/j.cgh.2019.06.031, 31252190

[41] Calder PC. Marine omega-3 fatty acids and inflammatory processes: effects, mechanisms and clinical relevance. Biochim Biophys Acta. (2015) 1851:469–84. doi: 10.1016/j.bbalip.2014.08.010, 25149823

[42] Müller S Quast T Schröder A Hucke S Klotz L Jantsch J . Salt-dependent chemotaxis of macrophages. PLoS One. (2013) 8:e73439. doi: 10.1371/journal.pone.0073439, 24066047 PMC3774673

[43] Wang X Lang F Liu D. High-salt diet and intestinal microbiota: influence on cardiovascular disease and inflammatory bowel disease. Biology. (2024) 13:674. doi: 10.3390/biology13090674, 39336101 PMC11429420

[44] Hwang S Yi HC Hwang S Jo M Rhee KJ. Dietary salt administration decreases Enterotoxigenic *Bacteroides fragilis* (ETBF)-promoted tumorigenesis via inhibition of colonic inflammation. Int J Mol Sci. (2020) 21:8034. doi: 10.3390/ijms21218034, 33126615 PMC7663446

[45] Ye P Xi Y Huang Z Xu P. Linking obesity with colorectal Cancer: epidemiology and mechanistic insights. Cancers. (2020) 12:1408. doi: 10.3390/cancers12061408, 32486076 PMC7352519

[46] Qi H Xia D Xu X. Dietary glycemic index, glycemic load, and renal cancer risk: findings from prostate, lung, colorectal, and ovarian cancer trial. Front Nutr. (2023) 10:1073373. doi: 10.3389/fnut.2023.1073373, 37346909 PMC10279873

[47] Freedman LS Schatzkin A Midthune D Kipnis V. Dealing with dietary measurement error in nutritional cohort studies. J Natl Cancer Inst. (2011) 103:1086–92. doi: 10.1093/jnci/djr189, 21653922 PMC3143422

[48] Brookhart MA Patrick AR Dormuth C Avorn J Shrank W Cadarette SM . Adherence to lipid-lowering therapy and the use of preventive health services: an investigation of the healthy user effect. Am J Epidemiol. (2007) 166:348–54. doi: 10.1093/aje/kwm070, 17504779

