# Supplementary material for: The healthy eating index 2020 and colorectal cancer risk: a prospective study based on the PLCO cohort
Source: Front Nutr. 2026 Jun 11;13:1793362. doi: 10.3389/fnut.2026.1793362 (PMC13293790; doi:10.3389/fnut.2026.1793362)
Supplement: Supplementary file 1 [file Table_1.docx]

**SUPPLEMENTARY MATERIAL**

**The Healthy Eating Index 2020 and Colorectal Cancer Risk: A Prospective Study Based on the PLCO Cohort**

**List of Supporting Information:**

**Supplementary Table 1.** Distribution of variables with missing data before and after imputation.

**Supplementary Table 2.** Fine–Gray competing-risk regression analysis for the association between HEI-2020 and CRC-specific mortality.

**Supplementary Table 3.** Subgroup analyses between HEI-2020 and CRC incidence.

**Supplementary Table 4.** Subgroup analyses between HEI-2020 and CRC mortality.

**Supplementary Table 5.** The sensitivity analyses between HEI-2020 and CRC incidence.

**Supplementary Table 6.** The sensitivity analyses between HEI-2020 and CRC mortality.

**Supplementary Table 1**. Distribution of variables with missing data before and after imputation.

| **Variable** | **Before imputation** | **After imputation** | **Number (%) with missing data** |
| --- | --- | --- | --- |
| **Race** |  |  | 37(0.04%) |
| white | 93986 (92.44%) | 94023 (92.44%) |  |
| non-white | 7686 (7.56%) | 7686 (7.56%) |  |
| **Marriage** |  |  | 193(0.19%) |
| married | 79595 (78.41%) | 79788 (78.45%) |  |
| unmarried | 21921 (21.59%) | 21921 (21.55%) |  |
| **Diabetes history** |  |  | 538(0.53%) |
| no | 94369 (93.28%) | 94907 (93.31%) |  |
| yes | 6802 (6.72%) | 6802 (6.69%) |  |
| **Aspirin use history** |  |  | 444(0.44%) |
| no | 53483 (52.81%) | 53927 (53.02%) |  |
| yes | 47782 (47.19%) | 47782 (46.98%) |  |
| **Family history of colorectal cancer** |  |  | 781(0.77%) |
| no | 88129 (87.32%) | 88910 (87.42%) |  |
| yes | 10306 (10.21%) | 10306 (10.13%) |  |
| possibly | 2493 (2.47%) | 2493 (2.45%) |  |
| **Diverticulitis/Diverticulosis history** |  |  | 643(0.64%) |
| no | 94243 (93.25%) | 94886 (93.29%) |  |
| yes | 6823 (6.75%) | 6823 (6.71%) |  |
| **Colorectal comorbidities history** |  |  | 897(0.89%) |
| no | 99456 (98.65%) | 100353 (98.67%) |  |
| yes | 1356 (1.35%) | 1356 (1.33%) |  |
| **Colorectal polyps history** |  |  | 626(0.62%) |
| no | 94318 (93.31%) | 94944 (93.35%) |  |
| yes | 6765 (6.69%) | 6765 (6.65%) |  |
| **Hypertension history** |  |  | 513(0.51%) |
| no | 68165 (67.36%) | 68678 (67.52%) |  |
| yes | 33031 (32.64%) | 33031 (32.48%) |  |
| **Family history of cancer** |  |  | 288(0.28%) |
| no | 44588 (43.96%) | 44876 (44.12%) |  |
| yes | 56833 (56.04%) | 56833 (55.88%) |  |
| **Smoking status** |  |  | 20(0.02%) |
| no | 48542 (47.74%) | 48562 (47.75%) |  |
| current/former | 53147 (52.26%) | 53147 (52.25%) |  |
| **Body mass index at baseline (kg/m2)** | 27.23±4.82 | 27.22±4.79 | 1348(1.34%) |
| **Weight fluctuation**^a^ | 2.88±0.76 | 2.88±0.76 | 1348(1.34%) |
| **Smoking pack-years** | 17.83±26.69 | 17.65±26.59 | 1163(1.16%) |
| **Daily cigarette consumption** |  |  | 124(0.12%) |
| 0 | 48542 (47.78%) | 48666 (47.85%) |  |
| 1-20 | 33203 (32.68%) | 33203 (32.65%) |  |
| >20 | 19840 (19.53%) | 19840 (19.51%) |  |

Note: Descriptive statistics are presented as (mean ± standard deviation) and number (percentage) for continuous and categorical.

^a^ Weight fluctuation defined as the participant's baseline weight minus weight at age 20.

**Supplementary Table 2**. Fine–Gray competing-risk regression analysis for the association between HEI-2020 and CRC-specific mortality.

| **Quartiles of HEI-2020** | **CRC-specific deaths** ^a^ | **Non-CRC deaths** | **Person-years** | **Unadjusted SHR** ^b^ **(95% CI)** | **Model 1** ^c^ **SHR (95% CI)** | **Model 2** ^d^ **SHR (95% CI)** |
| --- | --- | --- | --- | --- | --- | --- |
| Quartile 1 | 105 | 8,907 | 371,196.3 | 1.00 (reference) | 1.00 (reference) | 1.00 (reference) |
| Quartile 2 | 77 | 8,366 | 379,914.9 | 0.73 (0.54–0.98) | 0.72 (0.54–0.97) | 0.74 (0.55–0.99) |
| Quartile 3 | 68 | 7,767 | 386,608.6 | 0.65 (0.48–0.88) | 0.64 (0.47–0.88) | 0.67 (0.49–0.91) |
| Quartile 4 | 64 | 7,206 | 394,961.2 | 0.61 (0.44–0.83) | 0.59 (0.43–0.81) | 0.64 (0.46–0.88) |
| P for trend |  |  |  | <0.001 | <0.001 | 0.004 |
| ^a^ CRC-specific death was defined as death among participants with confirmed incident CRC for whom CRC was recorded as the underlying cause of death. Deaths from causes other than CRC were treated as competing events.  ^b^ SHR, subdistribution hazard ratio; CI, confidence interval.  ^c^ Model 1 was adjusted for age, sex, race, and marital status.  ^d^ Model 2 was adjusted for Model 1 plus hypertension, diabetes, smoking status, trial arm, daily cigarette consumption, alcohol drinking status, body mass index, aspirin use, family history of CRC, colorectal comorbidities, colorectal polyps, and diverticulitis/diverticulosis. | | | | | | |

**Supplementary Table 3**. Subgroup analyses between HEI-2020 and CRC incidence.

| **Variables** | **Number of participants** | | **Number of cases** | | **P_interaction_** |  | **HR ^b^ ( 95% confidence interval )** | | | **P_trend_ (Q4 vs Q1)** |
| --- | --- | --- | --- | --- | --- | --- | --- | --- | --- | --- |
|  |  |  |  |  |  | **Quartile 1** | **Quartile 2** | **Quartile 3** | **Quartile 4** |  |
| **Age(years)** |  |  | | 0.625 | |  |  |  |  |  |
| ≤65 | 71,841 | 642 | |  | | 1.00 (reference) | 1.03 (0.84, 1.26) | 0.84 (0.67, 1.04) | 0.84 (0.67, 1.06) | 0.144 |
| >65 | 29,868 | 458 | |  | | 1.00 (reference) | 0.91 (0.70, 1.18) | 0.85 (0.66, 1.11) | 0.89 (0.69, 1.16) | 0.629 |
| **Sex** |  |  | | 0.720 | |  |  |  |  |  |
| male | 49,459 | 608 | |  | | 1.00 (reference) | 1.02 (0.83, 1.26) | 0.84 (0.67, 1.05) | 0.91 (0.72, 1.15) | 0.445 |
| female | 52,250 | 492 | |  | | 1.00 (reference) | 0.88 (0.68, 1.14) | 0.76 (0.58, 1.00) | 0.94 (0.72, 1.22) | 0.072 |
| **Race** |  |  | | 0.472 | |  |  |  |  |  |
| white | 94,023 | 1,003 | |  | | 1.00 (reference) | 0.96 (0.81, 1.13) | 0.78 (0.65, 0.94) | 0.88 (0.74, 1.06) | 0.179 |
| non-white | 7,686 | 97 | |  | | 1.00 (reference) | 1.05 (0.61, 1.82) | 0.87 (0.49, 1.54) | 0.79 (0.61, 1.02) | 0.198 |
| **Marriage** |  |  | | 0.564 | |  |  |  |  |  |
| married | 79,788 | 855 | |  | | 1.00 (reference) | 1.01 (0.84, 1.21) | 0.79 (0.65, 0.96) | 0.90 (0.74, 1.09) | 0.277 |
| non-married | 21,921 | 245 | |  | | 1.00 (reference) | 0.83 (0.58, 1.18) | 0.82 (0.57, 1.16) | 0.77 (0.54, 1.10) | 0.156 |
| **Diabetes history** |  |  | | 0.629 | |  |  |  |  |  |
| no | 94,907 | 994 | |  | | 1.00 (reference) | 0.98 (0.83, 1.17) | 0.82 (0.69, 0.98) | 0.88 (0.73, 1.05) | 0.162 |
| yes | 6,802 | 106 | |  | | 1.00 (reference) | 0.84 (0.51, 1.39) | 0.55 (0.31, 0.96) | 0.73 (0.43, 1.27) | 0.267 |
| **Smoking status** |  |  | | 0.841 | |  |  |  |  |  |
| current/former | 53,147 | 615 | |  | | 1.00 (reference) | 0.97 (0.78, 1.19) | 0.81 (0.64, 1.01) | 0.81 (0.65, 1.03) | 0.082 |
| no | 48,562 | 485 | |  | | 1.00 (reference) | 0.99 (0.76, 1.27) | 0.78 (0.60, 1.02) | 0.93 (0.72, 1.20) | 0.566 |
| **Colorectal comorbidities history** |  |  | | 0.715 | |  |  |  |  |  |
| no | 100,353 | 1,084 | |  | | 1.00 (reference) | 0.96 (0.82, 1.13) | 0.81 (0.68, 0.96) | 0.85 (0.71, 1.02) | 0.077 |
| yes | 1,356 | 16 | |  | | 1.00 (reference) | 1.60 (0.44, 5.85) | 0.49 (0.09, 2.78) | 1.11 (0.26, 4.77) | 0.883 |
| **Diverticulitis/Diverticulosis history** |  |  | | 0.634 | |  |  |  |  |  |
| no | 94,886 | 1,022 | |  | | 1.00 (reference) | 0.97 (0.82, 1.14) | 0.81 (0.68, 0.96) | 0.85 (0.71, 1.02) | 0.077 |
| yes | 6,823 | 78 | |  | | 1.00 (reference) | 1.04 (0.56, 1.93) | 0.61 (0.3, 1.23) | 1.05 (0.56, 1.96) | 0.888 |
| **BMI** ^a^ |  |  | | 0.296 | |  |  |  |  |  |
| ≤30 | 78,562 | 833 | |  | | 1.00 (reference) | 0.89 (0.73, 1.07) | 0.73 (0.60, 0.89) | 0.80 (0.66, 0.97) | 0.021 |
| >30 | 23,147 | 267 | |  | | 1.00 (reference) | 1.23 (0.90, 1.68) | 0.97 (0.69, 1.37) | 1.00 (0.69, 1.45) | 0.999 |
| **Aspirin use history** |  |  | | 0.737 | |  |  |  |  |  |
| no | 53,927 | 605 | |  | | 1.00 (reference) | 0.99 (0.79, 1.23) | 0.85 (0.68, 1.08) | 0.90 (0.71, 1.13) | 0.367 |
| yes | 47,782 | 495 | |  | | 1.00 (reference) | 0.95 (0.75, 1.2) | 0.72 (0.55, 0.93) | 0.82 (0.64, 1.05) | 0.118 |
| **Family history of colorectal cancer** |  |  | | 0.860 | |  |  |  |  |  |
| no | 88,910 | 937 | |  | | 1.00 (reference) | 0.95 (0.80, 1.13) | 0.77 (0.64, 0.92) | 0.86 (0.71, 1.03) | 0.100 |
| yes/possibly | 12,799 | 163 | |  | | 1.00 (reference) | 1.07 (0.70, 1.63) | 0.92 (0.59, 1.42) | 0.87 (0.55, 1.39) | 0.569 |
|  |  |  | |  | |  |  |  |  |  |

^a^ BMI was defined as body mass index at baseline (kg/m²).

^b^ Hazard ratio was adjusted for age (years), sex (male, female), race (white and non-white), marital status (married, unmarried), history of diabetes (yes, no), smoking status (never, currently/ever), history of colorectal comorbidities (yes, no), history of colorectal diverticulitis/diverticulosis (yes, no), body mass index (kg/m²), aspirin use (yes, no), and family history of CRC (yes, no).

**Supplementary Table 4**. Subgroup analyses between HEI-2020 and CRC mortality.

| **Variables** | **Number of participants** | | **Number of cases** | | **P_interaction_** |  | **HR ^b^ ( 95% confidence interval )** | | | **P_trend_ (Q4 vs Q1)** |
| --- | --- | --- | --- | --- | --- | --- | --- | --- | --- | --- |
|  |  |  |  |  |  | **Quartile 1** | **Quartile 2** | **Quartile 3** | **Quartile 4** |  |
| **Age(years)** |  |  | | 0.340 | |  |  |  |  |  |
| ≤65 | 71,841 | 168 | |  | | 1.00 (reference) | 0.79 (0.53, 1.19) | 0.74 (0.49, 1.14) | 0.79 (0.51, 1.22) | 0.290 |
| >65 | 29,868 | 146 | |  | | 1.00 (reference) | 0.68 (0.44, 1.04) | 0.58 (0.37, 0.91) | 0.50 (0.31, 0.8) | 0.004 |
| **Sex** |  |  | | 0.874 | |  |  |  |  |  |
| male | 49,459 | 182 | |  | | 1.00 (reference) | 0.71 (0.49, 1.03) | 0.68 (0.46, 1.02) | 0.55 (0.35, 0.87) | 0.011 |
| female | 52,250 | 132 | |  | | 1.00 (reference) | 0.77 (0.47, 1.26) | 0.63 (0.38, 1.04) | 0.61 (0.38, 0.98) | 0.131 |
| **Race** |  |  | | 0.421 | |  |  |  |  |  |
| white | 94,023 | 281 | |  | | 1.00 (reference) | 0.73 (0.53, 0.99) | 0.61 (0.44, 0.85) | 0.64 (0.46, 0.90) | 0.009 |
| non-white | 7,686 | 33 | |  | | 1.00 (reference) | 0.76 (0.29, 1.99) | 0.96 (0.39, 2.36) | 0.44 (0.14, 1.36) | 0.154 |
| **Marriage** |  |  | | 0.895 | |  |  |  |  |  |
| married | 79,788 | 230 | |  | | 1.00 (reference) | 0.77 (0.55, 1.08) | 0.69 (0.48, 0.99) | 0.62 (0.42, 0.91) | 0.014 |
| non-married | 21,921 | 84 | |  | | 1.00 (reference) | 0.64 (0.35, 1.15) | 0.56 (0.3, 1.03) | 0.61 (0.34, 1.09) | 0.097 |
| **Diabetes history** |  |  | | 0.938 | |  |  |  |  |  |
| no | 94,907 | 285 | |  | | 1.00 (reference) | 0.73 (0.54, 1.00) | 0.64 (0.46, 0.88) | 0.62 (0.44, 0.86) | 0.005 |
| yes | 6,802 | 29 | |  | | 1.00 (reference) | 0.70 (0.25, 1.96) | 0.76 (0.28, 2.06) | 0.58 (0.19, 1.72) | 0.323 |
| **Smoking status** |  |  | | 0.603 | |  |  |  |  |  |
| current/former | 53,147 | 180 | |  | | 1.00 (reference) | 0.69 (0.47, 1.01) | 0.60 (0.40, 0.90) | 0.49 (0.31, 0.76) | 0.002 |
| no | 48,562 | 134 | |  | | 1.00 (reference) | 0.82 (0.51, 1.33) | 0.75 (0.46, 1.22) | 0.83 (0.51, 1.34) | 0.442 |
| **Colorectal comorbidities history** |  |  | | 0.070 | |  |  |  |  |  |
| no | 100,353 | 310 | |  | | 1.00 (reference) | 0.71 (0.52, 0.95) | 0.66 (0.48, 0.89) | 0.61 (0.44, 0.84) | 0.003 |
| yes | 1,356 | 4 | |  | | 1.00 (reference) | / | / | / | 1.000 |
| **Diverticulitis/Diverticulosis history** |  |  | | 0.513 | |  |  |  |  |  |
| no | 94,886 | 296 | |  | | 1.00 (reference) | 0.71 (0.53, 0.97) | 0.67 (0.49, 0.92) | 0.60 (0.43, 0.84) | 0.003 |
| yes | 6,823 | 18 | |  | | 1.00 (reference) | 1.03 (0.31, 3.46) | 0.34 (0.06, 1.79) | 0.86 (0.24, 3.11) | 0.203 |
| **BMI** ^a^ |  |  | | 0.151 | |  |  |  |  |  |
| ≤30 | 78,562 | 232 | |  | | 1.00 (reference) | 0.63 (0.44, 0.89) | 0.63 (0.44, 0.89) | 0.50 (0.35, 0.73) | <0.001 |
| >30 | 23,147 | 82 | |  | | 1.00 (reference) | 1.07 (0.62, 1.87) | 0.67 (0.35, 1.30) | 1.05 (0.56, 1.96) | 0.890 |
| **Aspirin use history** |  |  | | 0.797 | |  |  |  |  |  |
| no | 53,927 | 171 | |  | | 1.00 (reference) | 0.83 (0.56, 1.24) | 0.71 (0.47, 1.09) | 0.65 (0.42, 1.02) | 0.061 |
| yes | 47,782 | 143 | |  | | 1.00 (reference) | 0.62 (0.40, 0.97) | 0.58 (0.37, 0.92) | 0.57 (0.36, 0.90) | 0.017 |
| **Family history of colorectal cancer** |  |  | | 0.304 | |  |  |  |  |  |
| no | 88,910 | 265 | |  | | 1.00 (reference) | 0.66 (0.47, 0.92) | 0.64 (0.46, 0.90) | 0.62 (0.44, 0.88) | 0.007 |
| yes/possibly | 12,799 | 49 | |  | | 1.00 (reference) | 1.11 (0.55, 2.23) | 0.68 (0.30, 1.52) | 0.51 (0.20, 1.28) | 0.153 |
|  |  |  | |  | |  |  |  |  |  |

^a^ BMI was defined as body mass index at baseline (kg/m²).

^b^ Hazard ratio was adjusted for age (years), sex (male, female), race (white and non-white), marital status (married, unmarried), history of diabetes (yes, no), smoking status (never, currently/ever), history of colorectal comorbidities (yes, no), history of colorectal diverticulitis/diverticulosis (yes, no), body mass index (kg/m²), aspirin use (yes, no), and family history of CRC (yes, no).

**Supplementary Table 5**. The sensitivity analyses between HEI-2020 and CRC incidence.

| **Categories** | **HR** ^a^ **(Quartile 4 vs Quartile 1, 95% CI)** | **p for trend** |
| --- | --- | --- |
| Exclude Patients diagnosed within 2 years of follow-up | 0.83 (0.68,1.00) | 0.009 |
| Exclude extreme BMI ^b^ | 0.85 (0.72,1.01) | 0.017 |
| Replace the Indicator of cigarettes smoked ^c^ | 0.87 (0.73,1.03) | 0.024 |
| Excluding patients with diverticulitis/diverticulosis or colorectal  co-morbidity ^d^ | 0.85 (0.70,1.02) | 0.029 |

^a^ Hazard ratio was adjusted for age (years), sex (male, female), race (white and non-white), marital status (married, unmarried), history of hypertension (yes, no), history of diabetes (yes, no), smoking status (never, currently/ever), trial arm (intervention, control), number of cigarettes smoked (0, 1-20, > 20 cigarettes/day), history of alcohol consumption (yes, no), body mass index (kg/m²), aspirin use (yes, no), family history of colorectal cancer (yes, no), history of colorectal comorbidities (yes, no), history of colorectal polyps (yes, no), and history of colorectal diverticulitis/diverticulosis (yes, no).

^b^ BMI defined as body mass index at baseline (kg/m^2^).

^c^ Adjusting for pack-years of smoking (continuous) instead of daily cigarette consumption (0, 1-20, or >20).

^d^ Colorectal co-morbidity: Ulcerative colitis, Crohn's disease, Gardner's syndrome, or familial polyposis.

**Supplementary Table 6**. The sensitivity analyses between HEI-2020 and CRC mortality.

| **Categories** | **HR** ^a^ **(Quartile 4 vs Quartile 1, 95% CI)** | **p for trend** |
| --- | --- | --- |
| Exclude Patients diagnosed within 2 years of follow-up | 0.61 (0.44,0.84) | 0.001 |
| Exclude extreme BMI ^b^ | 0.60 (0.44,0.83) | 0.001 |
| Replace the Indicator of cigarettes smoked ^c^ | 0.62 (0.45,0.86) | 0.002 |
| Excluding patients with diverticulitis/diverticulosis or colorectal  co-morbidity ^d^ | 0.55 (0.39,0.79) | 0.001 |

^a^ Hazard ratio was adjusted for age (years), sex (male, female), race (white and non-white), marital status (married, unmarried), history of hypertension (yes, no), history of diabetes (yes, no), smoking status (never, currently/ever), trial arm (intervention, control), number of cigarettes smoked (0, 1-20, > 20 cigarettes/day), history of alcohol consumption (yes, no), body mass index (kg/m²), aspirin use (yes, no), family history of colorectal cancer (yes, no), history of colorectal comorbidities (yes, no), history of colorectal polyps (yes, no), and history of colorectal diverticulitis/diverticulosis (yes, no).

^b^ BMI defined as body mass index at baseline (kg/m^2^).

^c^ Adjusting for pack-years of smoking (continuous) instead of daily cigarette consumption (0, 1-20, or >20).

^d^ Colorectal co-morbidity: Ulcerative colitis, Crohn's disease, Gardner's syndrome, or familial polyposis
